# Differential expression of pro-inflammatory cytokines IL-15Ralpha, IL-15, IL-6 and TNFalpha in synovial fluid from Rheumatoid arthritis patients

**DOI:** 10.1186/s12891-015-0516-3

**Published:** 2015-03-12

**Authors:** Alicia Santos Savio, Ana Cecilia Machado Diaz, Araceli Chico Capote, Jamilet Miranda Navarro, Yunier Rodríguez Alvarez, Ricardo Bringas Pérez, Miguel Estévez del Toro, Gerardo E Guillen Nieto

**Affiliations:** Pharmaceutical Division, Center for Genetic Engineering and Biotechnology, Havana, CP 10600 Cuba; Rheumatology Department, H. Ameijeiras Hospital, San Lazaro 701, Havana, Cuba; Bioinformatics Department, Center for Genetic Engineering and Biotechnology, Havana, CP 10600 Cuba

**Keywords:** Cytokines, Rheumatoid arthritis, Osteoarthritis, Synovial fluid

## Abstract

**Background:**

Pro-inflammatory cytokines are directly implicated in the pathogenesis of Rheumatoid arthritis (RA). Variable clinical response to cytokine targeted therapies as TNFalpha and IL-6, strongly highlights the heterogeneity of inflammatory process in RA. Another cytokine, IL-15 has also been related to the inflammatory process in RA. Recently we described for the first time, the presence of its specific receptor, IL-15Ralpha, in synovial fluid (SF). The aim of this work was to compare the expression profile of IL-15Ralpha, its ligand IL-15, TNFalpha and IL-6 and how these cytokines are correlated in SF from RA patients taking as a reference Osteoarthritis (OA), an articular but not autoinmmune disease.

**Methods:**

Synovial fluids were obtained from the knee joints of 60 patients, 30 with confirmed diagnosis of RA and 30 with OA diagnosis. The levels of TNFalpha, IL-6, IL-15 and IL-15Ralpha were measured by ELISA. A statistical analysis was performed with GraphPad Prism v5.0 using the Mann–Whitney U test and Spearman’s rank correlation. A cluster analysis was run in MeV software v4.9.0 and differences across clusters were evaluated by an ANOVA including post-test analysis.

**Results:**

We found higher and significant levels of TNFalpha, IL-6 and IL-15Ralpha but not of IL-15 in RA compared with the OA group. Additionally, a high inter-individual variability in the levels of these 4 cytokines was observed in RA, although we identified 4 patients’ subgroups by cluster analysis of cytokines concentration in SF. We also found a positive correlation between IL-15Ralpha-IL-6 and IL-15Ralpha-IL-15, but not for other pairs of cytokines in RA. In addition we found correlation between the value of IL-15Ralpha in SF and disease activity score, DAS28.

**Conclusions:**

In our current work we found a high inter-individual variability in the levels of TNFalpha, IL-6, IL-15 and IL-15Ralpha in SF of RA patients and were identified four principal clusters of cytokines concentration in SF, suggesting the importance of identifying disease subset of patients for personalized treatment. Finally, we found a correlation between IL-15Ralpha-IL-6, IL-15Ralpha-IL-15, but we did not find any correlation between other pairs of studied cytokines in SF.

## Background

RA is a chronic autoimmune and inflammatory disease affecting synovial tissue in multiple joints. Pro-inflammatory cytokines are directly implicated in the pathogenesis of RA promoting synovial proliferation, hyperplasia and angiogenesis. This perpetuates inflammation, stimulates pannus formation and damage of bone and cartilage [1,2]. The proinflammatory cytokines interleukin 1 (IL1), tumour necrosis factor alpha (TNFalpha), and interleukin 6 (IL6), in particular, have been identified and intra-articular as well as systemic levels of these cytokines may to some extent reflect disease activity [3-6]. Treatment with anti TNF alpha inhibitors has been very effective as a biological therapy [7]. Unfortunately, 40% of RA patients who receive a TNF inhibitor fail to achieve an adequate response [8]. Other biological therapies including Tocilizumab [9,10], rituximab [11] and abatacept [12] have been successful in the treatment of RA, but the variable clinical response to these targeted therapies strongly highlights the heterogeneity of inflammatory process in RA. Another cytokine, IL-15 has also been related to the inflammatory process in RA. IL-15 is a pro-inflammatory cytokine which acts at the innate and acquired immune response [13]. This cytokine recruits circulating memory T cells in the synovial membrane and may up regulate other pro-inflammatory cytokines [14,15]. These events include induction of TNF production through activation of synovial T cell and by macrophages via a cell contact dependent mechanism [16], also the activation of Th17 lymphocytes driving IL17 production [17]. Moreover, soluble IL-15 appears to be an important contributor to osteoclastogenesis promoting bone erosion [18]. IL-15 mediates its biological effects by binding to a high affinity heterotrimeric receptor complex which is comprised of IL-15Ralpha, IL-2/15Rbeta and IL-Rgamma. IL-15Ralpha is a unique high affinity private subunit for IL-15. IL-15Ralpha may be secreted as a functional soluble molecule (s-IL-15Ralpha) and could behave as antagonist or agonist by forming a complex with IL-15 [19-21], also, it has been described that soluble IL-15Ralpha could bind to membrane bound IL-15 to induce a reverse signaling mechanism up-regulating the production of IL-6, IL-8, and TNFalpha by human monocytes and in the cancer cell line PC-3 [22,23]. These antecedents highlight the importance of this receptor in IL-15 biology and trigger previous studies of IL-15Ralpha in synovial fluids, where we have detected increasing levels of this receptor in synovial fluids from RA regards to OA [24]. The aim of this work is to compare the expression profile of IL-15Ralpha, its ligand IL-15 and two validated cytokines targets in RA, TNF alpha and IL-6, in SF from RA regards to Osteoarthritis (OA) patients. In addition, we will assess inter-individual expression of these cytokines and will analyze how this cytokines are correlated.

## Methods

### Patients and samples

Synovial fluids were obtained from the knee joints of 60 patients, 30 with a confirmed diagnosis of RA based on DAS28 criteria and 30 with a diagnosis of OA. All patients showed inflammation and abundant synovial fluid in the cavities of synovial joints. This study was approved by the Amejeiras Hospital Ethics committee and all patients gave written informed consent. Patient demographics are listed in Table 1. Synovial fluid was directly aspirated from the inflamed joint and collected in tubes, immediately after we added hyaluronidase type IV (H3884, Sigma, USA) at 10 ug/mL to synovial fluid, and mixed by inversion followed by spinning at 1000 g for 10 min within 30 min of sample collection. The acellular portion of synovial fluid (synovial liquid) was stored at −80°C before subsequent analysis.

**Table 1:** **Patient’s data**

**Patients**	**RA (n = 30)**	**OA (n = 30)**
**Sex (M/F)**	6/24	11/19
**Age (years)**	52 ^+^- 15	62 ^+^- 12
**Disease duration (years)**	12^+^- 11	6 ^+^- 10
**Rheumatoid factor (+/−)**	15/15	–
**DAS28**	4.27^+^- 1.23	–
**ERS**	39 + −26.3	
**CRP**	17.5 + −15.6	
**DMARD (MTX)**	30	–

RA, rheumatoid arthritis; OA Osteoarthritis; DAS28, disease activity score in 28 joints; ERS, erythrocyte sedimentation rate; (CRP) C reactive protein; DMARD, disease-modifying antirheumatic drug; MTX, metotrexate.

### Cytokine measurement

Levels of cytokines TNFalpha, IL-6, e IL-15 in SF were measured in duplicate using commercially available ELISA kits purchased from R&D Systems according to the manufacturer’s instructions (Quantikine Human TNF alpha, DTA00C(detection limit was 15.6 pg/mL), Quantikine Human IL-6, D6050 (detection limit was 3.12 pg/mL), Quantikine Human IL-15, D1500 (detection limit was 3.9 pg/mL). IL-15Ralpha was quantified by ELISA as previously described [25]. Briefly, the 96-well microtiter plates (Costar, Corning Inc., NY, USA) were treated with 2% glutaraldehyde solution for 2 h at 37°C. After two washes with water, plates were coated with 10 μg/mL of P8 peptide/well, and the plates were then incubated at 4°C overnight. After three washes with phosphate buffered saline pH 7.4 (PBS) containing 0.05% Tween 20, nonspecific binding sites were blocked by incubation for 1 h at 37°C in PBS containing 1% BSA. The blocking solution was replaced by samples (synovial liquid diluted 2-fold in PBS, containing 0.01% BSA and 0.05% Tween 20 or different concentration of recombinant IL-15Ralpha-Fc (147-IR, R&D) in the same buffer). All the samples were in triplicate. Following incubation at 37°C for 2 h, we did three washes with PBS containing 0.05% Tween 20. IL-15Ralpha was detected with a specific antibody against IL-15Ralpha (AF247, R&D System). The bound IL-15Ralpha was detected with HRP-conjugated goat antihuman IgG (A0170, Sigma, USA) by incubation at 37°C for 1 h, followed by 5 washes with PBS, 0.1% Tween 20. The reaction was visualized by adding the substrate solution (3,3,5,5-tetramethylbenzidine [TMB]), and absorbance at 450 nm was measured with an ELISA plate reader (Biotrak GE, Healthcare, USA). The detection limit was 0.25 nM.

### Statistical analysis

Statistical analyses were carried out using GraphPad Prism v5.0 (GraphPad Software, San Diego, CA, USA) software implementation of Mann Whitney U-test and Spearman’s rank correlation. For each RA patient the expression ratio relative to the averaged expression of OA patients was calculated. Hierarchical cluster analysis (HCL) of log_2_ expression ratios profiles are based on Spearman’s rank correlation and average linkage was performed with MeV analysis software v4.9.0 [26]. Analysis of variance (ANOVA) between clusters was calculated using the Kruskal-Wallis test with Dunn’s post-test analysis. For all analysis, P < 0.05 was taken as significant.

## Results

### Baseline characteristics of patients

Baseline clinical data of patients are presented in Table 1. RA patients received treatment with oral methotrexate (MTX) and low-dose prednisone. All patients were naive to biological agents. The OA patients were a more heterogeneous group regarding treatment, some received Glucosamine sulfate, some analgesic or physiotherapies. Both groups of patients were heterogeneous in terms of age and disease duration. Regarding sex, there was a higher number of women in the RA group, ratio 4:1 female/male according to what has been reported for this disease.

### Comparison of cytokines IL-15Ralpha, IL-15, IL-6, and TNFalpha levels in synovial fluid from RA and OA patients

**Figure 1:**
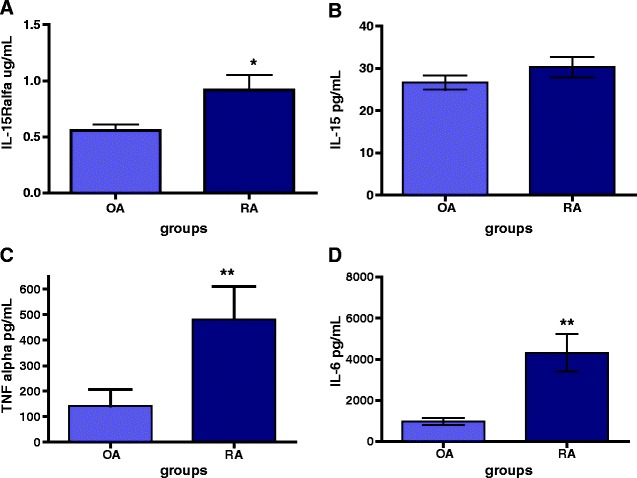
**Measured levels of sIL-15Ralpha, IL-15, IL-6 and TNFalpha in synovial fluids from RA and OA patients.** Cytokine concentration of IL-15Ralpha **(A)**, IL-15 **(B)**, TNFalpha **(C)** and IL-6 **(D)** were measured in in duplicate SF samples from RA (n = 30) and OA (n = 30) by ELISA. Box plot represents media ± SD. Differences between two groups were performed by Mann–Whitney *U* test for nonparametric data.

Quantification by ELISA of cytokines IL-15Ralpha, IL-15, IL-6, and TNFalpha in synovial fluids from the knee joints of 30 RA and 29 OA patients, showed that SF from RA patients contained significantly higher concentrations of IL-15Ralpha (p = 0.03, Figure 1A), TNFalpha (p = 0.002, Figure 1C) and IL-6 (p = 0.001, Figure 1D) than the OA group, but in the case of IL-15 we did not find any significant difference between RA and OA groups (p = 0.29) (Figure 1B).

### Cluster analysis of SF from RA patients

**Figure 2:**
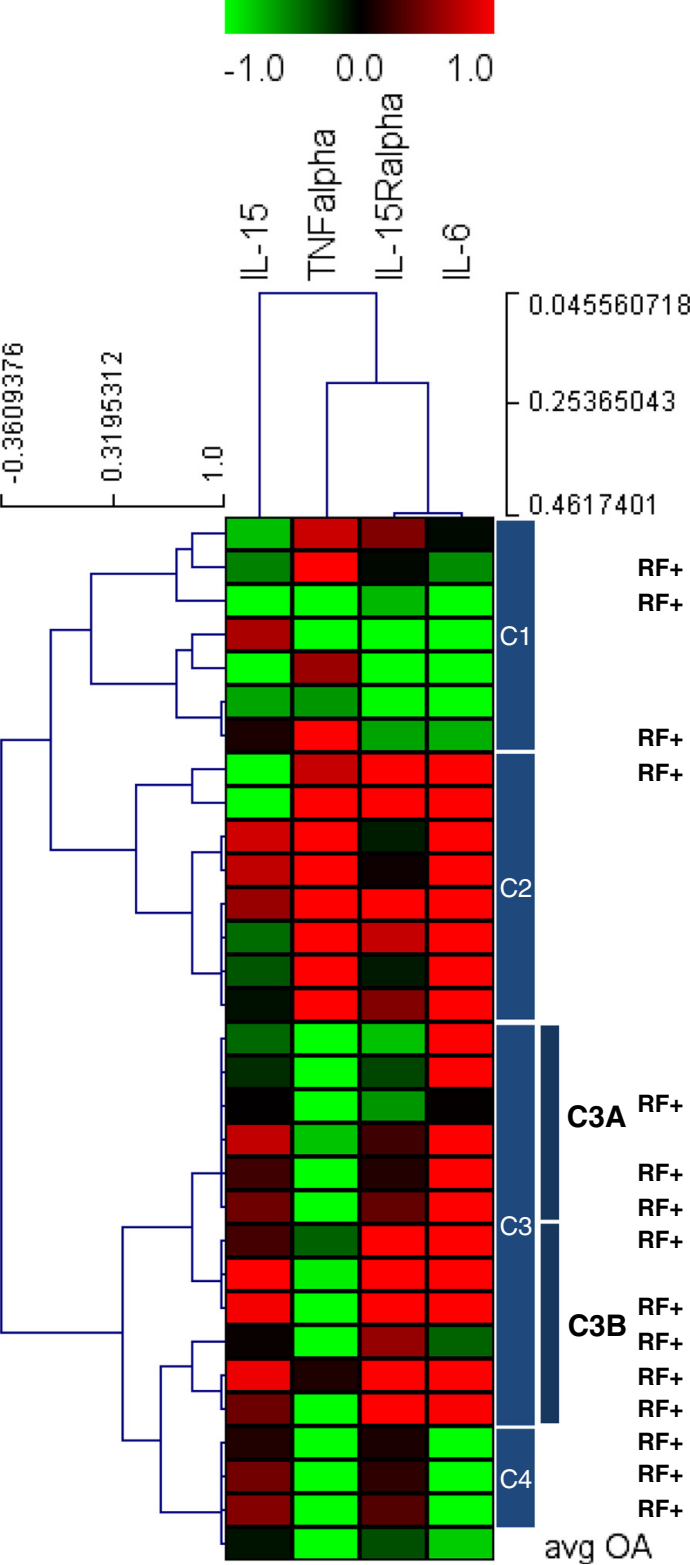
**Cluster analysis of cytokines concentration in SF from RA patients.** Hierarchical Cluster analysis of cytokines expression profiles obtained for 30 RA patients (Spearman rank correlation and single linkage algorithm). Dendrogram resulting from clustering the RA SFs (rows) identified four principal groups of patients (C1-C4). The most intense red blocks represent highly over-expressed cytokines and most intense green blocks represent the cytokines with greatest degree of under-expression. Black indicates cytokines whose expression is similar in the AR and OA reference samples. At the right side C3A and C3B denote two subclusters of cluster 3 showing different levels of IL-15Ralpha expression. RF+ indicates patients which are positive to Rheumatoid factor.

Two-dimensional Hierarchical cluster analysis of expression patterns in SF of the four studied cytokines was performed (Figure 2). Four principal clusters of patients were identified and also a high correlation between IL-15Ralpha and IL-6 cytokines. Comparison of the SF cytokine concentrations in each cluster was carried out using the Kruskal-Wallis test with Dunn’s post-test analysis. Cluster 1 includes 7 patients which showed low levels of all cytokines (more significantly IL-6 and IL-15Ralpha) or only high level in one of them; Cluster 2 includes 8 patients expressing significantly high concentration of TNFalpha (C2vs.C3:P < 0.001; C2 vs.C4:P < 0.05) and IL-6 (C2vs.C1:P < 0.001; C2vs.C4:P < 0.01) and most of them express high or mid concentration of IL-15Ralpha and/or IL-15. Cluster 3 includes 12 patients expressing significantly low levels of TNFalpha (C3vs.C2:P < 0.001), significantly high levels of IL-6 (C3vs.C1:P < 0.05) and IL-15Ralpha (C3vs.C1:P < 0.05) and high levels of IL-15. This cluster includes 8 out of 15 patients RF+. Cluster 4 includes 3 patients expressing significantly low levels of TNFalpha (C4vs.C2:P < 0.05) and IL-6 (C4vs.C2:P < 0.01), and positive to IL-15, IL-15Ralpha and RF. Analysis of variance showed significant differences across clusters, which may be indicative of heterogeneity of the inflammation process.

### Correlation studies among all four cytokines in RA

**Figure 3:**
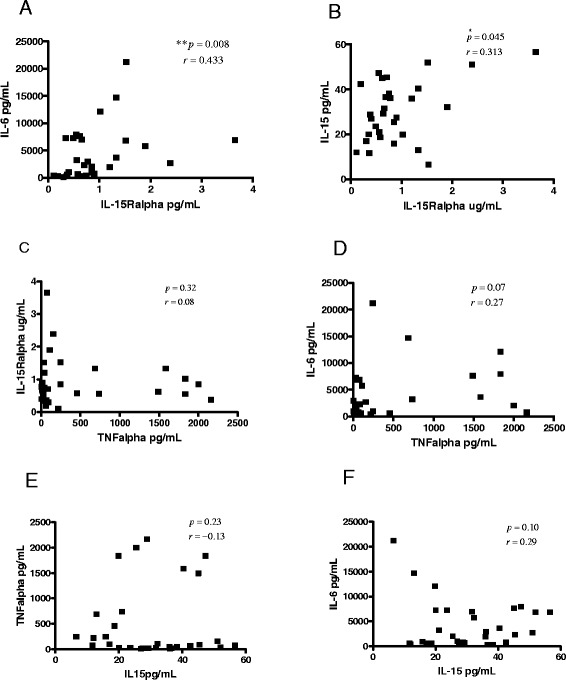
**Correlation between cytokines concentration in SF from RA patients.** Correlations among concentrations of IL-15Ralpha, IL-15, IL-6 and TNF alpha in SF from RA patients were analyzed using spearman’s rank correlation for all possible combinations. Correlations were significant between IL-6/ IL-15Ralpha **(A)** and IL-15/Il-15Ralpha **(B),** but not for IL-15Ralpha/TNFalpha **(C)**, IL-6/TNFalpha **(D)**, TNFalpha/IL-15 **(E)** or IL-6/IL-15 **(F)**. r (the Spearman correlation coefficient), p (p value, was set at significant at 0.05).

**Figure 4:**
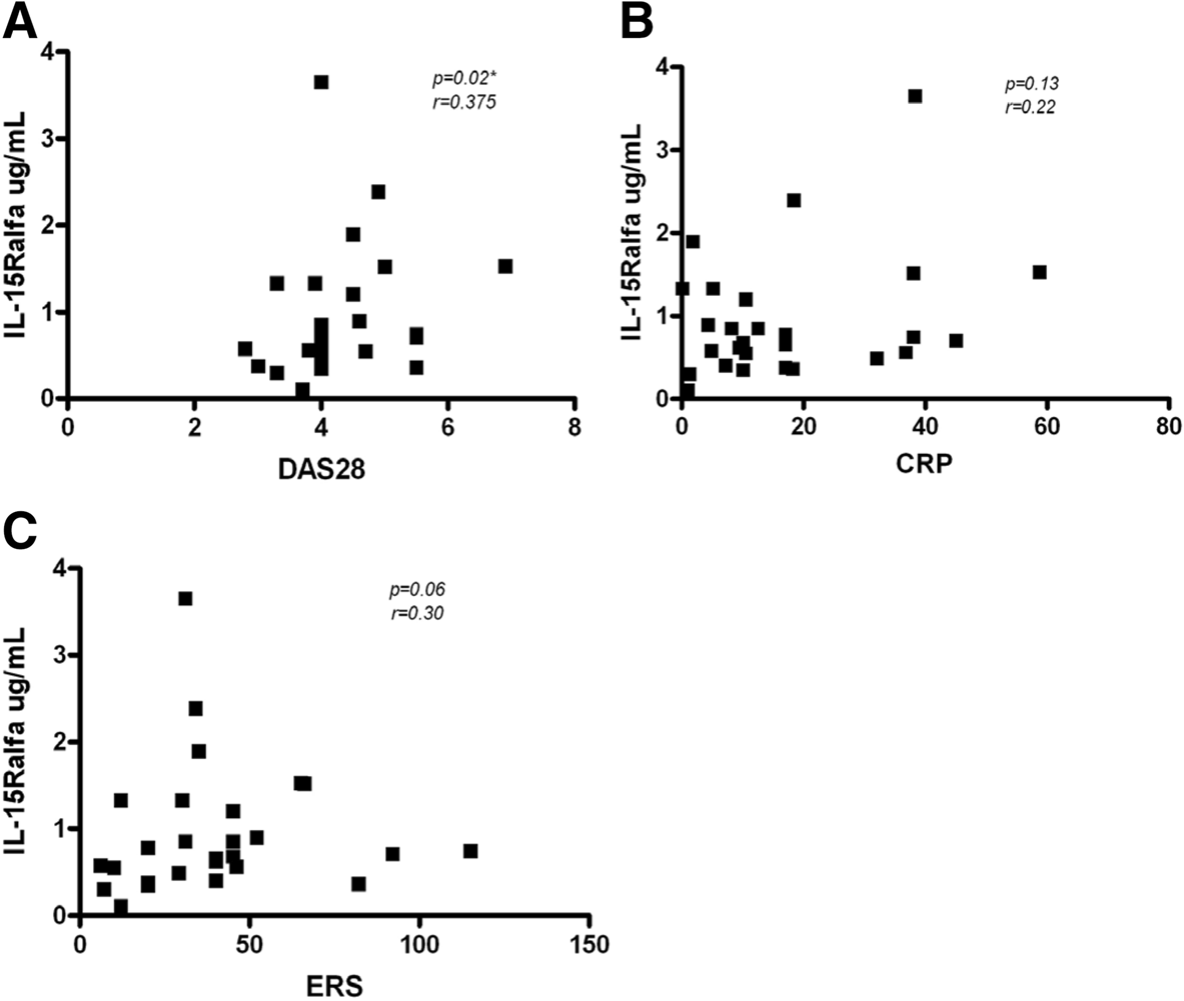
**Correlation between IL-15Ralpha in SF from RA patients and ERS, PCR and DAS28.** Correlations of IL-15Ralpha concentration and DAS28, CRP and ERS were analyzed using spearman’s rank correlation. Correlations were significant IL-15Ralpha and DAS28 **(A)**, but not for CRP and ERS **(B and C)**.

Additionally, we performed correlation analysis among all studied cytokines. In correspondence with our previous report, we found a correlation between IL-15Ralpha and IL-6 (r = 0.4335, p = 0.008, Figure 3A). In current work we also found a correlation between IL-15Ralpha and IL-15 (r = 0.313, p = 0.01, Figure 3B). However, there was no positive correlation between IL-15Ralpha and TNFalpha (r = 0.08, p = 0.32) IL-15 and TNFalpha (r = −0.136, p = 0.23), IL-15 and IL-6 (r = 0.10, p = 0.29) or TNFalpha-IL-6 (r = 0.27, p = 0.07) (Figure 3C-F). Also we did the correlation analysis among values of IL-15Ralpha, DAS28, ERS and CRP for each patient at the moment for taking the sample. As a result we found a correlation between IL-15Ralpha and DAS28, but neither to ESR nor CRP. (Figure 4).

## Discussion

Due to RA is a syndrome comprising different pathogenic subsets, studying cytokine expression profile in inflammatory SF provides a unique opportunity to examine the microenvironment within the inflamed joint. In our current work we assessed expression of IL-15Ralpha, IL-15, IL-6 and TNFalpha in RA compared to OA, a rheumatic non autoimmune disease. Besides, we examined how expression of these cytokines inters individual’s patients.

In correspondence to previous studies, we found high varied levels of TNFalpha and IL-6 in patients with RA which were significant compared to the OA group [27-29]. Presence of soluble IL-15Ralpha has been less studied in RA, but it was previously detected as soluble receptor in serum from mice and humans [30]; recently we detected soluble IL-15Ralpha in SF from RA and OA patients, which was the highest in RA. In current work, we confirmed these results with a higher number of patients. It is important to highlight that the ELISA performed to detect IL-15Ralpha used as a capture a previously described peptide which specifically binds to IL-15Ralpha and displace IL-15/IL-15Ralpha binding in a dose dependent manner [25]. Therefore, we considered that detected IL-15Ralpha could not form complexes with endogenous IL-15.

Taking into account presence of IL-15Ralpha in synovial fluid, we were interested in to know how IL-15Ralpha is correlated to its ligand IL-15 and TNFalpha, an important proinflammatory cytokine validated as a therapeutic target in RA. We considered previous reports showing that IL-15 induces TNFalpha production by synovial cells. Also we measured IL-6 based on our previous report where we found correlation between IL-15Ralpha and L-6.

Regarding IL-15, we did not find significant differences for soluble IL-15 comparing RA and OA groups. Interestingly, we found high levels of IL-15 in 6 patients within OA group which corresponds to patients with early diagnosis of disease. Biological onset of OA is not clearly understood but evidence suggests that progression of cartilage degradation is mediated largely by pro-inflammatory cytokines [31,32]. Recent data suggest that innate immunity may be a driver of the OA process, thus, increased levels of the IL-15 protein are found in the synovial fluid of early knee OA patients when compared to end-stage OA [33]. More recently it was reported that serum IL-15 levels are associated with severity of pain in patients with knee OA; this suggests that IL-15 might play a role in the pathogenesis of early OA [34]. Disease duration was not considered as a variable in our study at the moment of including patients. Actually, in our study 60% of patients (18/29) with OA were 24 or fewer months from diagnosis which could explain the fact that we did not find significant differences in IL-15 between RA and OA groups, conversely as previously reported elsewhere [35,36].

Additionally, we studied the inter-individual cytokine expression in RA patients. A pronounced inter-individual diversity of expression patterns for these 4 cytokines was observed in SF. This finding corresponds to previously reported results of heterogeneity obtained when IL-6, TNF alpha, IL-1alpha and IL-1beta which were determined by immunohistochemistry in synovial tissue of RA patients [37]. In current work, we identified 4 clusters of SFs corresponding to different patterns of concentration of these cytokines. Cluster 1 groups 7 patients with low levels of cytokines concentrations. Cluster 2 group patients with at least 3 increased cytokines, high levels of TNFalpha and IL-6 were observed in all patients included in this cluster. Additionally IL-15 or IL-15Ralpha was elevated, suggesting a high inflammation level. Cluster 3 includes patients negative to TNFalpha with high levels of IL-6 which could have high or mid levels of IL-15Ralpha and IL-15. Cluster 4 include patients with high or mid levels of IL-15 and IL-15Ralpha which are negative to IL-6 and TNF alpha, suggesting that the IL-15 system could play an important role in the inflammatory process in these patients, independent of IL-6 or TNFalpha.

Indeed, we found a correlation between IL-15Ralpha and DAS28 suggesting a relation between IL-15Ralpha level and disease activity. Other finding was that 11 of 15 patients positive RF are included in clusters 3 and 4. It is in agreement with a previous report of association between IL-15 and RF. So far, it has not been reported any association between IL-15Ralpha and RF.

Regarding disease duration, we could stratify patients into 2 groups being 1st group (n = 9) those with early disease (we consider early disease patients with <2 years from time of diagnosis); the 2nd group (n = 13) comprises those patients > 10 years from time of diagnosis. Both groups were statistically analyzed and no differences were found. There was none association between disease duration and IL-15Ralpha. Interestingly, we found that in C3B, one of the two subgroups of cluster 3 and including patients with high level of IL-15Ralpha, 83% of them had late disease.

Heterogeneity observed in the expression pattern of the studied cytokines should be taken into account when cytokines are used as targets in a therapeutic approach. In our current study, Cluster 3 and Cluster 4, which include 15 of 30 patients with signs of inflammation, are negative to TNFalpha. This could explain the fact that even when inhibiting TNFα in patients with active RA which results in a rapid and sustained improvement of their signs and symptoms of disease, it has been reported that more than 40% of them do not respond to this treatment [38]. In patients treated with Tocilizumab a humanized monoclonal antibody against IL-6 receptor, also has been reported about 40% of inadequate response [39]. Previous studies have shown a positive correlation between TNFalpha levels in serum and response to therapies inhibiting TNFalpha, although there are reports with the opposite result [40-42]. Frequently, cytokine levels in serum do not correspond with cytokine levels present in synovial fluid.

Cluster analysis has also shown a positive correlation between IL-15Ralpha and IL-6. Additionally we performed correlation analysis between the four studied cytokines. Correlation between IL-15Ralpha and IL-6 was confirmed and interestingly, we found also correlation between IL-15Ralpha and IL-15, which could be indicative of IL-15/IL-15Ralpha complexes detected by ELISA However, we did not find any correlation between IL-15 and IL-6 which suggested a mechanism of IL-6 induction by IL-15Ralpha independently of soluble IL-15. Also IL-15 and TNFalpha were none correlated, probably because induction of TNFalpha by IL-15 takes place in the microenvironment of target cells depending on cell contact and it is not reflected in acellular synovial fluid. In our experiments, we found a well-defined group of patients being negative to TNFalpha; however these patients express high or medium levels of IL-15, only 3 patients showed both TNF alpha and IL-15 high. We did not find any correlation between other pairs of cytokines in this work (IL-15Ralpha/TNFalpha and TNFalpha/IL-6). Therefore, these results reinforce our suggestion that IL-15 reverse signaling through membrane IL-15, could take place in RA synovium as other inducing pathway of IL-6 [24]. Nevertheless, it is important to take into account complexities of the IL-15/IL-15R system, three different functional forms of IL-15 have been identified: 1) the soluble cytokine, 2) IL-15R-independent membrane-bound IL-15 and 3) membrane-anchored IL-15 through IL-15Rα [13,20,23]. Also, IL-15Rα may be secreted as a functional soluble molecule (s-IL-15Rα) and could form a complex with IL-15 [19,21]. Thus, these results raise another question about the role of different presentation forms and complexities of cytokines/cytokines receptor system highlighting the need of personalized medicine in treatment using the cytokines as a target.

## Conclusions

In our current work, we found higher levels of TNFalpha, IL-6 and IL-15Ralpha in RA than OA, but not of IL-15 suggesting that IL-15 could be implicated in the OA pathology. Also, our results confirm heterogeneity of the inflammatory process assessed in RA patients where high inter-individual diversity expression patterns of IL-15Ralpha, IL-15, IL-6 and TNFalpha were observed. Despite this, we have identified 4 main clusters of concentration of these cytokines in SF suggesting the importance of identifying disease subgroups to personalize treatment. Finally, we found correlations between IL-15Ralpha-IL-6 and IL-15Ralpha-IL-15, but we did not find any correlation between other pairs of studied cytokines, highlighting a role for IL-15Ralpha in the RA pathology.

## Abbreviations

IL-6: Interleukin- 6
IL-15: Interleukin-15
IL-15Ralpha: Interleukin 15receptor alpha
TNFalpha: Tumor necrosis factor alpha
IL-2: Interleukin-2
IL-1: Interleukin-1
RA: Rheumatoid arthritis
OA: Osteoarthritis
SF: Synovial fluid
DAS28: Disease Activity Score 28
ELISA: Enzyme-linked immunosorbent assay
ANOVA: Analysis of Variance
BSA: Bovine serum albumin
PBS: Phosphate buffered saline
MTX: Methotrexate
